# Unbiased high-content screening reveals Aβ- and tau-independent synaptotoxic activities in human brain homogenates from Alzheimer’s patients and high-pathology controls

**DOI:** 10.1371/journal.pone.0259335

**Published:** 2021-11-08

**Authors:** Hao Jiang, Thomas J. Esparza, Terrance T. Kummer, David L. Brody

**Affiliations:** 1 Department of Neurology, Washington University School of Medicine, St Louis, Missouri, United States of America; 2 Henry M Jackson Foundation for the Advancement of Military Medicine, Bethesda, Maryland, United States of America; 3 National Institute of Neurological Disorders and Stroke, Bethesda, Maryland, United States of America; 4 Department of Neurology, Uniformed Services University of the Health Sciences, Bethesda, Maryland, United States of America; Istituto di Ricerche Farmacologiche Mario Negri IRCCS, ITALY

## Abstract

Alzheimer’s disease (AD) is tightly correlated with synapse loss in vulnerable brain regions. It is assumed that specific molecular entities such as Aβ and tau cause synapse loss in AD, yet unbiased screens for synaptotoxic activities have not been performed. Here, we performed size exclusion chromatography on soluble human brain homogenates from AD cases, high pathology non-demented controls, and low pathology age-matched controls using our novel high content primary cultured neuron-based screening assay. Both presynaptic and postsynaptic toxicities were elevated in homogenates from AD cases and high pathology non-demented controls to a similar extent, with more modest synaptotoxic activities in homogenates from low pathology normal controls. Surprisingly, synaptotoxic activities were found in size fractions peaking between the 17–44 kDa size standards that did not match well with Aβ and tau immunoreactive species in these homogenates. The fractions containing previously identified high molecular weight soluble amyloid beta aggregates/”oligomers” were non-toxic in this assay. Furthermore, immunodepletion of Aβ and tau did not reduce synaptotoxic activity. This result contrasts with previous findings involving the same methods applied to 3xTg-AD mouse brain extracts. The nature of the synaptotoxic species has not been identified. Overall, our data indicates one or more potential Aβ and tau independent synaptotoxic activities in human AD brain homogenates. This result aligns well with the key role of synaptic loss in the early cognitive decline and may provide new insight into AD pathophysiology.

## Introduction

Alzheimer disease (AD) is a progressive, neurodegenerative condition characterized by a prolonged decline in cognitive abilities. It is the most prevalent late-life cognitive disease that affects an estimated 6.2 million Americans in 2021. The pathological hallmarks of AD are extracellular senile plaques (SP) which contain abundant amyloid-beta (Aβ) and intracellular neurofibrillary tangles (NFT) characterized by hyperphosphorylated tau protein. However, accumulated evidence suggests that SP and NFT are not limited to patients with AD but are also present in the brains of cognitively normal elders [1, 2]. In fact, many individuals are able to remain cognitively normal and endure high SP loads for decades [3, 4]. Exploring the characteristics of such individuals compared to those AD patients who had been clinically and histopathologically diagnosed is the subject of much recent interest [5–10], and may be of great importance for understanding AD pathogenesis. We and others have found that soluble Aβ aggregate/”oligomer” concentrations in demented AD cases are higher and more tightly correlated with Aβ plaque coverage compared with non-demented individuals with AD pathology [11]. Furthermore, significantly lower total Zn^2+^ levels and no detectable association of Aβ oligomers with post-synaptic terminals are found in these individuals [12]. On the other hand, Aβ42 monomer levels are higher in these cognitively normal individuals than in AD cases [13]. To date, the mechanisms underlying the apparent dissociation between clinical impairment and AD pathology remain unknown; it is possible that these asymptomatic individuals may possess ‘resilient’ factors or have substantial cognitive ‘reserve capacity’ that prevents progression of clinical impairment [14, 15]. It is also possible that they are merely at the initial, ‘preclinical’ stage of AD [10], or that these hallmark pathologies are truly dissociated from the clinical syndrome [16].

**Fig 1:**
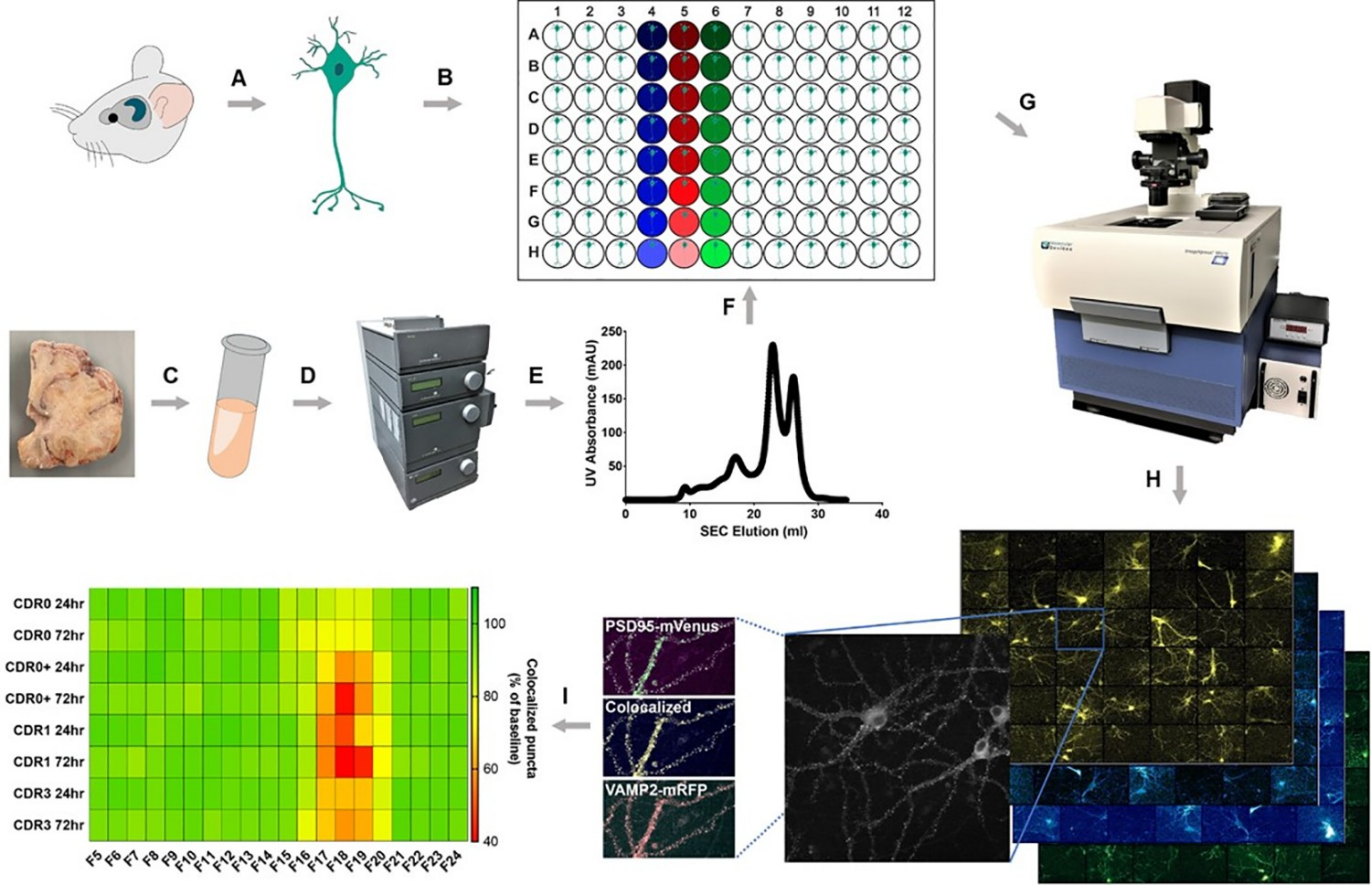
Schematic of the assessment of synaptic changes using the high-content imaging system. (A, B) Hippocampal neurons from genetically modified mice with fluorescent synapses were isolated and cultured in treated 96-well microplates for more than 20 days in vitro (DIV); (C-F) Human frontal cortex homogenates from control and AD samples were separated by size exclusion chromatography (SEC) and added to individual wells of 96 well plates; (G) Synapses were imaged before and after the addition of fractionated human brain samples using the ImageXpress high-content screening system equipped with environmental control unit for live cell imaging; (H) Pre-synaptic VAMP2-mRFP puncta, post-synaptic PSD95-mVenus puncta, and colocalized puncta were analyzed; (I) Heat map analysis showed synaptotoxic activities in SEC fractions from control and AD human samples at 24 and 72 hours; significant loss of colocalized synaptic puncta was identified in neurons exposed to low molecular weight (F17-20) fractions.

First described by Gonatas [17], there have been numerous studies demonstrating that AD is tightly correlated with synapse loss in vulnerable brain regions [18, 19], which has led to the hypothesis that loss of synapses is a key event in early cognitive decline. While the mechanism of synapse loss in AD is not fully understood, it is presumed that specific molecular entities, such as Aβ and tau are responsible for synaptic degeneration [20–22]. Unbiased screens, however, have not been performed. Evaluation of the nature of neuronal and synaptic changes in cognitively normal individuals with AD pathology and in AD cases may provide an advantage for identification of synaptotoxic substances and for understanding of the progression AD. We recently developed a robust high-content imaging method for assessing synaptic changes in a 96 well plate format [23]. Our method uses serial imaging of endogenous labeled presynaptic VAMP2-mRFP [24] and postsynaptic PSD95-mVenus [25] protein in long-term cultured murine primary neurons to quantitate the number of synaptic puncta for the assessment of synaptic changes (Fig 1). We previously showed that multiple synaptotoxic activities can be detected in size-exclusion chromatography (SEC) fractioned brain homogenates from 3xTg-AD mice [26]. Interestingly, both Aβ-related and apparently Aβ-independent synaptotoxic activities have been identified [23]. However, the synaptotoxicities in human brain homogenates have not been assessed in this fashion.

In an attempt to understanding the differences between AD pathology positive non-demented individuals and clinically affected AD cases, in this study we screened for synaptotoxic activities in SEC fractioned brain homogenates from a total of 29 individuals. In addition, we evaluated the role of Aβ and tau in synaptotoxic SEC fraction by immunodepletion.

## Materials and methods

### Human frontal cortical brain samples

Human frontal cortical tissue samples (n = 29) were obtained from the Charles F. and Joanne Knight Alzheimer’s Disease Research Center at Washington University School of Medicine in Saint Louis. Cognitive status was evaluated with a validated retrospective postmortem interview with an informant to establish the Clinical Dementia Rating (CDR). Cognitively normal subjects (CDR0), cognitively normal subjects with AD pathology (CDR0+), mildly demented subjects (CDR1), and severe demented subjects (CDR3) were used in this study (Table 1).

**Table 1:** Characteristics of human brain frontal cortex samples.

Sample ID	Gender	Age, yrs	Clinical Dementia Rating	Postmortem Interval, hrs
1	Male	89.6	CDR0	21.8
2	Female	72.1	CDR0	15.0
3	Male	91.1	CDR0	8.5
4	Male	97.0	CDR0+path	3.5
5	Female	91.7	CDR0+path	12.0
6	Female	100.9	CDR0+path	21.0
7	Female	95.4	CDR0+path	23.0
8	Male	80.8	CDR0+path	5.5
9	Female	76.7	CDR0+path	5.0
10	Female	86.4	CDR1	6.7
11	Female	89.0	CDR1	19.0
12	Female	92.7	CDR1	23.0
13	Female	94.2	CDR1	11.6
14	Female	104.4	CDR1	19.0
15	Male	68.6	CDR1	21.0
16	Female	86.0	CDR1	18.0
17	Female	81.0	CDR1	21.0
18	Male	72.7	CDR1	4.5
19	Female	76.8	CDR1	17.0
20	Male	64.6	CDR3	11.0
21	Male	75.1	CDR3	4.0
22	Male	85.9	CDR3	10.0
23	Female	80.8	CDR3	5.15
24	Female	85.8	CDR3	20.0
25	Male	80.6	CDR3	13.5
26	Male	71.6	CDR3	7.0
27	Male	81.0	CDR3	5.25
28	Male	86.6	CDR3	12.0
29	Female	81.5	CDR3	7.5

### Homogenization of brain tissue and immunodepletion of Aβ and tau

Approximately 200 mg of frozen human frontal cortical tissue was weighed and placed into ice-cold ‘Neurobasal Salt’ solution (homemade buffer containing all inorganic salts, D-Glucose, HEPES, and Sodium Pyruvate of Neurobasal medium) containing 1X protease inhibitor cocktail (Sigma-Aldrich) at 200 mg/mL and homogenized using a Dounce tissue grinder on ice as described before [27]. After centrifugation at 21,000 xg for 45 min at 4°C, the top 90% of supernatant was collected. Protein concentration was assessed with the Micro BCA Protein Assay Kit (ThermoFisher).

Immunodepletion of Aβ and tau was performed using 150 μg of total protein from soluble fraction of tissue homogenates. Five micrograms of each HJ3.4 and HJ5.1 (for Aβ) [28, 29] and HJ8.7 (anti-tau118-122 AAGHV) and HJ9.3 (anti-tau589-598 GGKVQIINKK) antibodies [30] were added and incubated at 4°C for 1 hour. Thirty microliters of BSA blocked Protein G PLUS-Agarose (Santa Cruz) was added to the sample and incubated at 4°C overnight on a rotator. Samples were then centrifuged at 3,000 xg for 5min at 4°C. The immunodepleted supernatant was collected.

### Size exclusion chromatography

One hundred fifty micrograms of total protein was injected into a 1 mL sample loop and separated on a Superdex 200 10/300 GL column eluted with 35 mL of ‘Neurobasal Salt’ solution supplemented with 1X Pen-Strep at a flow rate of 0.8 mL/min using an AKTA Purifier FPLC. Twenty-Eight 1 ml fractions that covered the entire UV 280 positive eluent were collected and stored at 4°C. All samples were tested within 2 days, and the rest were stored at -80°C for further analysis.

### Measurement of soluble Aβ1–40, 1–42, and oligomeric Aβ using sandwich ELISA

The amount of total Aβ and oligomeric Aβ were evaluated by sandwich ELISA as described previously [27]. In brief, 100 μL of an anti-Aβ HJ3.4 (for oligomeric Aβ) or HJ2 (for total Aβ1–40) and HJ7.4 (for total Aβ1–42) antibody was coated to 96-well Nunc MaxiSorp flat-bottom plates (ThermoFisher) at 20 μg/mL in carbonate buffer overnight and then blocked with 2% BSA in PBS for 1 hour at room temperature. Samples and standard were loaded and incubated overnight; 6M guanidine-HCl was added at 5% of total sample volume for the total Aβ measurement to prevent oligomerization during the incubation. Biotinylated HJ3.4 antibody in PBS at 100 ng/mL was used as detection antibody and incubated at room temperature for 1 hour for the measurement of both total and oligomeric Aβ. Poly-streptavidin HRP-20 (Fitzgerald) in PBS at 30 ng/mL was then added and incubated for 30 min at room temperature. After final wash, the assay was developed by adding 100 μL of 3,3′,5,5′-Tetramethylbenzidine (TMB) (Sigma-Aldrich) and the absorbance was read on a Synergy 2 plate reader (BioTek) at 650 nm.

### Measurement of total tau and phosphorylated tau (T181) using sandwich ELISA

The amount of total tau and phosphorylated tau (T181) were evaluated by sandwich ELISA using commercially available kits (ThermoFisher) following the manufacture instructions. In brief, 100 μL of samples and standards were loaded onto precoated plates and incubated overnight. After washing, 100 μL of biotinylated antibody Hu Tau (total) biotin conjugated solution was added and incubated for 2 hours at room temperature followed by incubation of 1X Streptavidin-HRP solution. After final washing, 100 μL of TMB was added and incubated for 30 minutes and the absorbance was read on a Synergy 2 plate reader (BioTek) at 650 nm. For phosphorylated tau (T181), the same procedure was used except 50 μL of antibody Hu Tau (pT181) and anti-rabbit IgG HRP was used.

### Measurement of A11 immunoreactive oligomer, total tau, and phosphorylated tau using indirect ELISA

The amount of A11 immunoreactive oligomer, total tau, and phosphorylated tau were also measured by indirect ELISA. In brief, 100 μL of total protein was coated to 96-well Nunc MaxiSorp flat-bottom plates at 20 μg/mL in sample buffer overnight at 4°C. Plate was then washed and blocked with 4% BSA for 1 hour at room temperature. One hundred micro-liters of anti-Oligomer A11 antibody (for Aβ and other oligomeric structure, ThermoFisher), HJ8.7 (anti-tau118-122), HJ9.3 (anti-tau589-598) [30], or anti-tau phospho T205 (for phosphorylated tau T205, Abcam) was added to the well at 1 μg/mL and incubated at 4°C overnight with shaking. After wash, HRP conjugated anti-mouse or anti-rabbit antibody (Cell Signaling Technolgies) was added to the well at 1:1000 dilution and incubated at room temperature for 2 hours with shaking. After final wash, the assay was developed by adding 100 μL of TMB (Sigma-Aldrich) and the absorbance was read on a Synergy 2 plate reader (BioTek) at 650 nm.

### Long-term primary neuron culture in 96-well microplates

All cell culture procedures were performed under standard aseptic working conditions. Ninety-six well glass bottom plates (Cellvis) were coated with 50 μg/mL Poly-D-lysine (PDL) (Sigma-Aldrich) at 50 μL per well at room temperature overnight. Plates were then washed with sterile distilled water three times and dried for at least 30 min before use. Hippocampal neurons were collected and cultured as described [23]. To minimize evaporation and edge effects on the microplate during imaging, the interwell region of the culture plate was filled with sterile water. Following 2 days *in vitro* (DIV), 50 μL of plating medium with 5 mM Cytosine β-D-arabinofuranoside (Ara-C) was added to the wells. At DIV 5, 50% of medium was replaced with maintenance medium containing 1X B27 Plus in Neurobasal Plus medium (ThermoFisher) with 100mM GlutaMAX. Thereafter, 50% of medium was replaced with fresh maintenance medium every 4 to 5 days for up to 30 days.

### Live primary neuron based high-content screening of synaptic activity

Live primary neuron based 96-well plate high-content screening was performed using MetaXpress High-Content Image Acquisition and Analysis Software 6.1 and ImageXpress Micro XLS Wide-field High-Content Analysis System equipped with temperature and CO_2_ environmental control units (Molecular Devices) and X-Cite 110LED white light LED light source (Excelitas) at 32 ± 2°C with 5% CO_2_. Images were taken with a Nikon 60X CFI Super Plan Fluor ELWD objective. For all samples, triplicated measurements were performed (n = 3 wells per SEC fraction). Five images per well were obtained by taking five horizontally adjacent imaging sites near the center of the well. Laser-based autofocusing methods on both plate bottom and well bottom were used. Exposure times of 400 ms and 1200 ms were used for VAMP2-mRFP and PSD95-mVenus respectively. To assess synapses in live primary neurons, cells were cultured for 21 days, then the baseline scans were performed at DIV 22. Potentially synaptotoxic substances were then added, and the same regions of each well were imaged twice. For these experiments, the second and third scans were performed 24 and 72 hours later to evaluate the acute to short-term effects of the treatments.

### Semi-automatic image analysis using Fiji/ImageJ and MetaXpress

Synaptic density was assessed by analyzing the total number of presynaptic (VAMP2-mRFP) puncta, postsynaptic (PSD95-mVenus) puncta, and colocalized puncta for each image. Images taken at different time points were semi-automatically aligned, processed, and analyzed using MetaXpress 6.1 and Fiji/ImageJ (V1.52n). All images were processed and analyzed automatically using macros with batch processing in Fiji/ImageJ and batch processing ‘journal’ function in MetaXpress 6.1. First, time-lapse images from the same image site taken at different time points were automatically aligned using MetaXpress. Overlapped regions of interest (ROI) from images taken at different time points were processed automatically using a batch process ‘journal’ including steps of Flatten background using fluorescent light (pixel size = 5), 2D Deconvolution using Nearest Neighbors method (Filter size = 10, Scaling factor = 0.97, Suppress noise checked), and Morphology Filters using Top-hat method (Area = 50 pixels^2^). All images were then processed automatically using a batch process macro in ImageJ including steps of AutoThreshold with MaxEntropy, filtered with medium method. Finally, the total number of synaptic puncta from each processed image were automatically counted using the ‘Analyze Particles’ function in Fiji/ImageJ. Particles between 2 and 50 voxels in size were counted. Last, to analyze colocalized synaptic puncta, processed VAMP2 and PSD95 images from step two were merged using ImageJ macro ‘Batch RG Merge’, and the merged images were analyzed automatically using batch process ‘Synapse Counter’ ImageJ Plugin [31] with 0.1 and 0.2 for ‘Rolling ball radius’ and ‘Maximum filter radius’, ‘Otsu’ for ‘Method for threshold adjustment’, and 2 to 50 voxel size was used for both pre- and postsynaptic particle size.

### Statistical analysis

All data were analyzed with Fiji/ImageJ, and statistical analysis was performed with Prism 7.0 (GraphPad Software). The total number of counted synaptic puncta from different time points were normalized to percentage baseline puncta number. The sample size for statistical analyses was the number of individual human brain specimens in each group. One-way ANOVA followed by Tukey’s multiple comparisons test was used for ELISA analysis among all sample groups. Two-way ANOVA followed by Dunnett’s multiple comparisons test was used to compare synapse loss in CDR0+, CDR1, and CDR3 sample groups with control CDR0 group, as well as to compare synapse loss in immunoprecipitated sample groups with no treatment control group. Two-way ANOVA followed by Tukey’s multiple comparisons test was used to compare synapse loss among all sample groups. A *P*-value ≤ 0.05 was considered statistically significant.

## Results

### Characterization of study subjects

Fresh frozen frontal cortical tissue from 29 human subjects were used in this study, including cognitively normal subjects with no AD pathology (‘CDR0’: mean age = 84.2 ± 8.6 years, post-mortem interval (PMI) = 15.1 ± 5.4 hours), cognitively normal subjects with Alzheimer’s pathology (‘CDR0+’: mean age = 90.4 ± 8.8 years, PMI = 11.7 ± 7.8 hours), mildly demented Alzheimer’s cases (‘CDR1’: mean age = 85.2 ± 10.2 years, PMI = 16.0 ± 6.0 hours), and severely demented Alzheimer’s cases (‘CDR3’: mean age = 79.4 ± 6.7 years, PMI = 9.5 ± 4.6 hours) (Table 1). All samples were obtained from the Charles F. and Joanne Knight Alzheimer Disease Research Center at Washington University in St Louis.

### Comparison of Aβ and tau levels in brain homogenates indicates differences among CDR0, CDR0+, and AD patients

**Fig 2:**
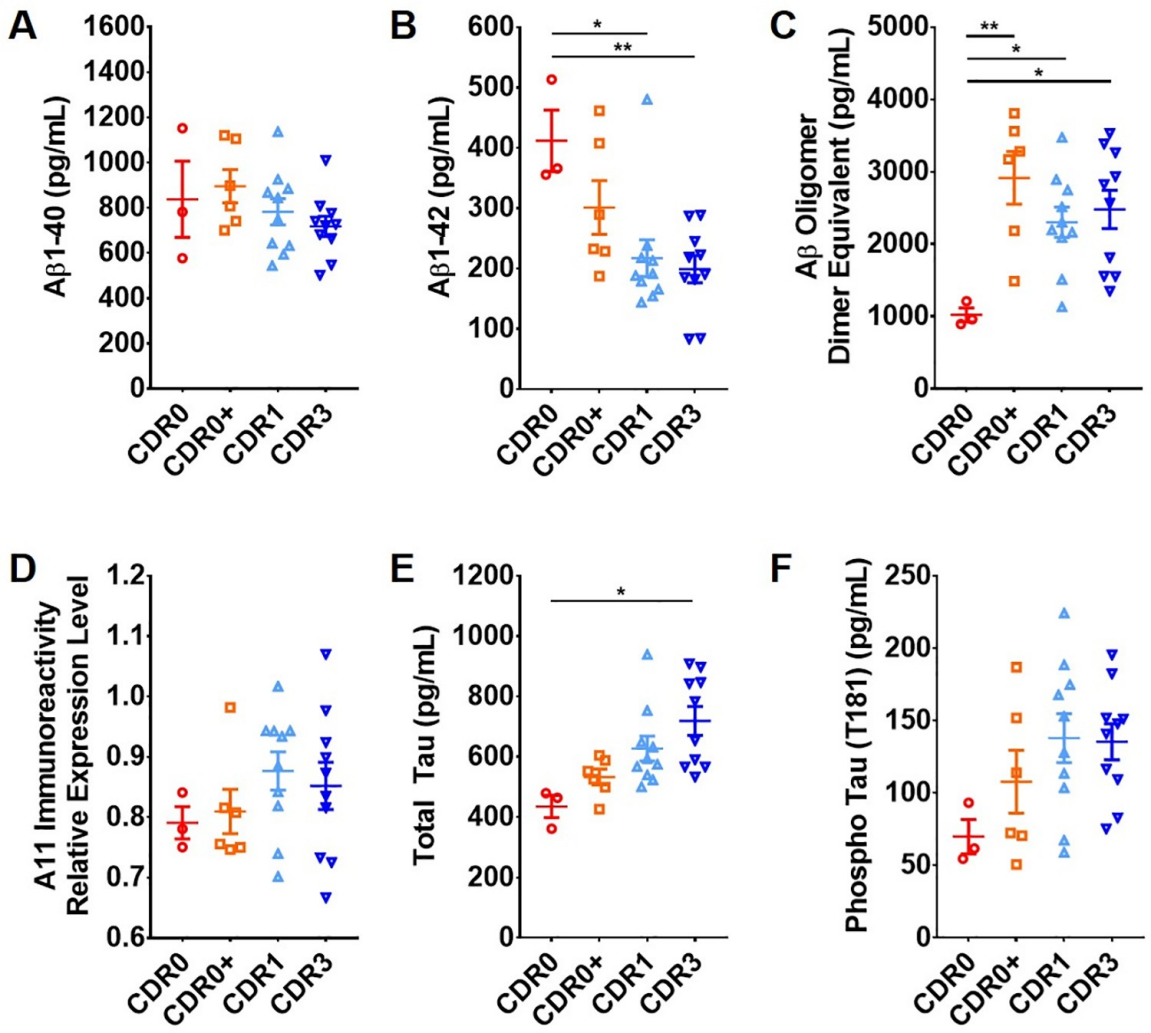
Scatterplots of individual levels of Aβ and tau in brain homogenates from control and AD cases. All samples were measured in triplicate. One-way ANOVA followed by Tukey’s multiple comparisons test were used for all measurements (*** *p* ≤ 0.001, ** *p* ≤ 0.01, * *p* ≤ 0.05, error bar indicates SEM). (A) The levels of Aβ_1–40_ did not differ among sample groups; (B) The level of Aβ_1–42_ in the CDR0 group was significantly higher in than in the CDR1 group (*p =* 0.016) and CDR3 group (*p =* 0.0078); (C) The level of Aβ oligomer measured by sandwich ELISA in CDR0 group was significantly lower than CDR0+ (*p =* 0.0021), CDR1 (*p =* 0.0352), and CDR3 (*p =* 0.0156) groups; (D) The levels of A11 positive oligomer measured by indirect ELISA did not significantly differ between groups; (E) The level of total tau was significantly lower in the CDR0 group than in the CDR3 group (*p =* 0.0101); (F) The levels of T181 phosphorylated tau did not significantly differ between groups.

We and others have demonstrated previously that the level of Aβ oligomers is tightly correlated with Aβ plaque coverage and higher in CDR1 patients than CDR0+ AD pathology controls [11]. In this study, we assessed the levels of several Aβ and tau species in soluble brain homogenates from all study subjects. The level of total soluble Aβ_1–40_ and Aβ_1–42_ in aqueous sample buffer was assessed by a highly sensitive ELISA assay [11]. Among all sample groups, Aβ_1–40_ showed no statistical difference (F(3,25) = 1.289, *p =* 0.2998) (Fig 2A), whereas Aβ_1–42_ levels in AD patients were significantly lower than CDR0 normal controls (Fig 2B) by one-way ANOVA (F(3,25) = 5.293, *p =* 0.0058) followed by Tukey’s multiple comparison test (CDR0 vs CDR1: *p =* 0.016 and CDR0 vs CDR3: *p =* 0.0078). These findings indicated the expected correspondence of soluble Aβ_1–42_ level and AD progression [32]. The level of Aβ_1–42_ in CDR0+ samples was intermediate. The level of soluble Aβ aggregates in aqueous sample buffer was assessed by our previously established sandwich [11]. There were significant differences between groups by one-way ANOVA (F(3,25) = 5.663, *p =* 0.0042). All subjects with AD pathology, including CDR0+, showed significantly higher levels of Aβ oligomers than normal controls (CDR0+: *p =* 0.0021, CDR1: *p =* 0.0352, and CDR3: *p =* 0.0156) (Fig 2C). In contrast, indirect ELISA using the A11 anti-oligomer antibody [33] revealed a trend towards higher level of A11 immunoreactivity in CDR1 and CDR3 subjects, but without a statistical difference between groups (F(3,25) = 0.8219, *p =* 0.4941) (Fig 2D). The level of total soluble tau and phosphorylated tau (T181) in aqueous sample buffer from all subjects was similarly compared. Total tau was significantly different between groups (F(3,25) = 4.412, p = 0.0127) with lower level of total tau in the CDR0 group compared with the CDR3 group (p = 0.0101) (Fig 2E). Phosphorylated T181 tau showed no significant difference between groups (F(3,25) = 2.078, *p =* 0.1287) (Fig 2F). Interestingly, indirect ELISA using the HJ8.7 (anti-tau118-122 AAGHV) antibody [30] revealed significantly higher tau in CDR0 group compared with all AD pathology positive samples (F(3,25) = 3.751, *p =* 0.0237; CDR0 vs CDR0+: *p =* 0.0484, CDR0 vs CDR1: *p =* 0.0005, and CDR0 vs CDR3: *p =* 0.0002) (S1 Fig) while no difference was found when using the HJ9.3 (anti-tau589-598 GGKVQIINKK) antibody [30] (F(3,25) = 1.004, *p =* 0.4075; S1 Fig). In addition, indirect ELISA using an anti-phosphorylated tau (phospho T205) antibody revealed a significantly higher level in the CDR0 group compared with CDR1 (F(3,25) = 3.448, *p =* 0.0318; CDR0 vs CDR1: *p =* 0.0408) (S1 Fig).

### Aβ and tau in soluble lysates migrate at multiple sizes in SEC fractions

**Fig 3:**
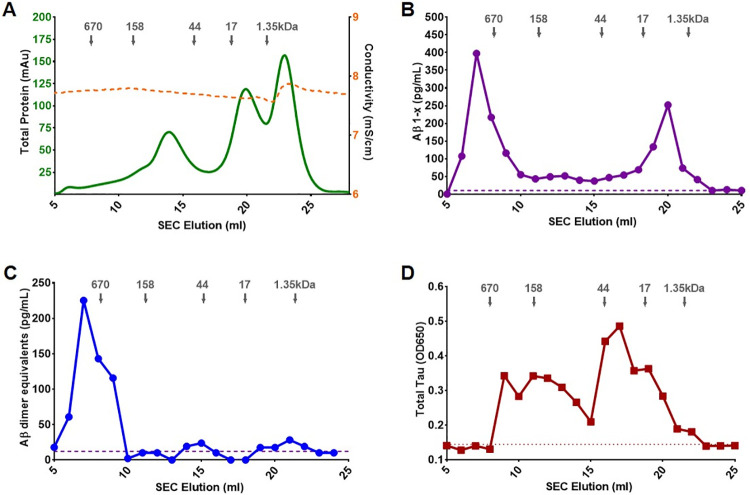
Representative SEC profile of total protein, conductivity, total Aβ level, oligomeric Aβ level, and total tau levels from a CDR3 patient. Total protein and conductivity were measured by UV detector (mAu) and conductivity detector (mS/cm); the levels of total Aβ, oligomeric Aβ, and tau in each fraction were measured by ELISA. (A) SEC elution profile of total protein and conductivity; (B) High molecular weight Aβ aggregates/oligomers (MW ≥ 670 kDa) was detected in Fractions 6 to 9; low molecular weight Aβ (MW ≤ 17 and ≥ 1.35 kDa) was detected in Fractions 18 to 22; no other major peak was measured in other fractions, however, very low signals just above the lower detection limit (dashed line) were identified in intermediate fractions; (C) High molecular weight Aβ aggregates/oligomer (MW ≥ 670 kDa) was detected in Fractions 6 to 9, no other major peak was measured in the other fractions; (D) Total tau was detected in Fractions 9 to 22; the largest level of total tau was detected in Fractions 16 to 17 with an estimated MW around 44 kDa.

To understand synaptotoxic activity in SEC fractions, we first measured the level of total Aβ, oligomeric Aβ, tau, total protein, and salt concentration in samples, which may independently cause synapse loss in our assay (Fig 3). Total protein concentration from each fraction was evaluated by UV280 measurement. Most proteins eluted in fractions 14 to 17 mL, 20 to 22 mL, and 23 to 25 mL. The concentration of total salt was estimated by conductivity measurement. The level of total salt in all collected fractions were nearly identical (Fig 3A). Total Aβ and oligomeric Aβ level in each SEC fraction were assessed by sandwich ELISA. For total Aβ, two major peaks were present centered at 6 to 9 mL (high molecular weight) and 18 to 21 mL (low molecular weight) of total eluent with estimated molecular weights of larger than 670 kDa and slightly higher than 1.35 kDa. Very low levels of Aβ, close to the detection limit, were distributed between the two peaks (Fig 3B) [34]. For oligomeric Aβ, high molecular weight Aβ oligomer (MW ≥ 670 kDa) was detected in Fractions 6 to 9, no other major peak was identified (Fig 3C). The level of total tau was assessed by indirect ELISA. Tau immunoreactivity was distributed widely from Fraction 9 to 22. The highest amount of tau was found in the 16–17 mL fractions, with an estimated molecular weight in the 44 kDa range (Fig 3D).

### HCS screening reveals synaptotoxic activities in low molecular weight SEC fractions

**Fig 4:**
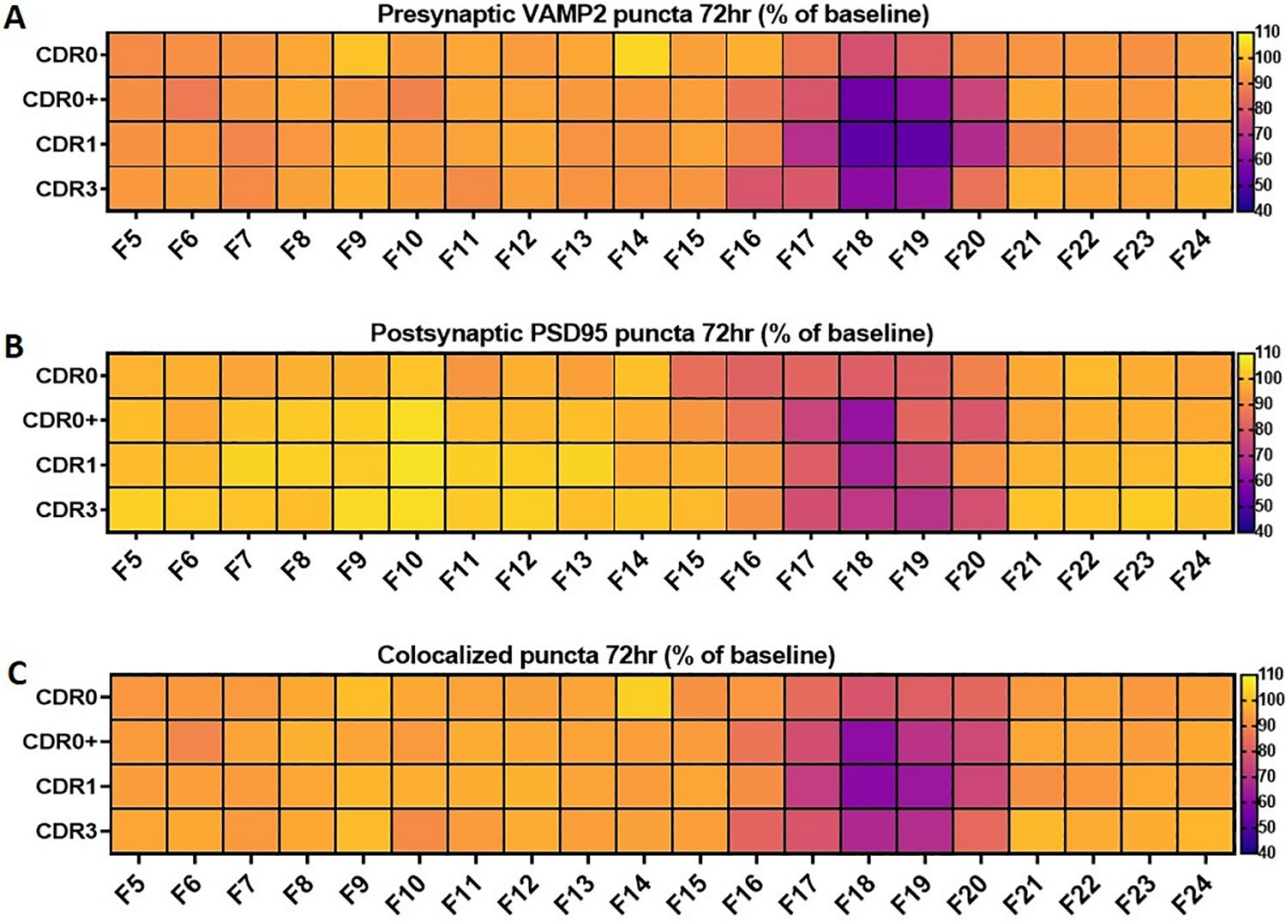
Heatmaps of synaptotoxic activities in each SEC fractions from control and AD case groups at 72 hours. Scatterplots of synaptotoxic activities of each individual subject is shown in S3 Fig. (A) Comparison of presynaptic VAMP2 synaptotoxic activities among control and AD case groups at 72 hours; significant VAMP2 loss were found in wells incubated with lysate fractions 17 to 20; several fractions from CDR0+ and CDR1 showed statistically more VAMP2 loss than from CDR0; (B) Comparison of postsynaptic PSD95 synaptotoxic activities among control and AD case groups at 72 hours. Loss of PSD95 post synaptic puncta was found in wells incubated with lysate fractions 17 to 20. Fraction 18 from CDR0+ and CDR1 brain homogenates caused more PSD95 postsynapse loss than fraction 18 from CDR0 brains. Fraction 19 from CDR3 brain homogenates caused more PSD95 postsynapse loss than fraction 19 from CDR brains. (C) Comparison of colocalized pre and post synaptic puncta among control and AD case groups at 72 hours; loss of colocalized synaptic puncta were found in wells incubated with fractions 17 to 20. Fractions 17–19 from CDR0+ and CDR1 brain homogenates caused more colocalized synaptic puncta loss than comparable fractions from CDR0 brains.

Interestingly, synaptotoxic activities were identified in all samples including CDR0 normal controls (Figs 1I and 4). Human brain SEC fractions that showed synaptotoxic activities were distributed from 17 to 20 mL of the total SEC eluent, which indicates larger molecular weight components than the synaptotoxic fractions identified in 3xTg-AD mouse brain lysates assessed under identical conditions (fractions 20–22) [23]. Furthermore, the fractions with synaptic toxicity were slightly larger than those containing Aβ monomer (fractions 18–21, Fig 3B), and smaller than the peak of the tau distribution (fractions 16–17, Fig 3C). The fractions with synaptic toxicity had relatively low total protein concentrations and similar conductivity (Fig 3A) relative to other non-synaptotoxic fractions from the same brains.

### SEC fractions from CDR0+ and CDR1 brains showed the most severe synaptotoxic activities while fractions from CDR0 brains showed the mildest synapse loss

**Fig 5:**
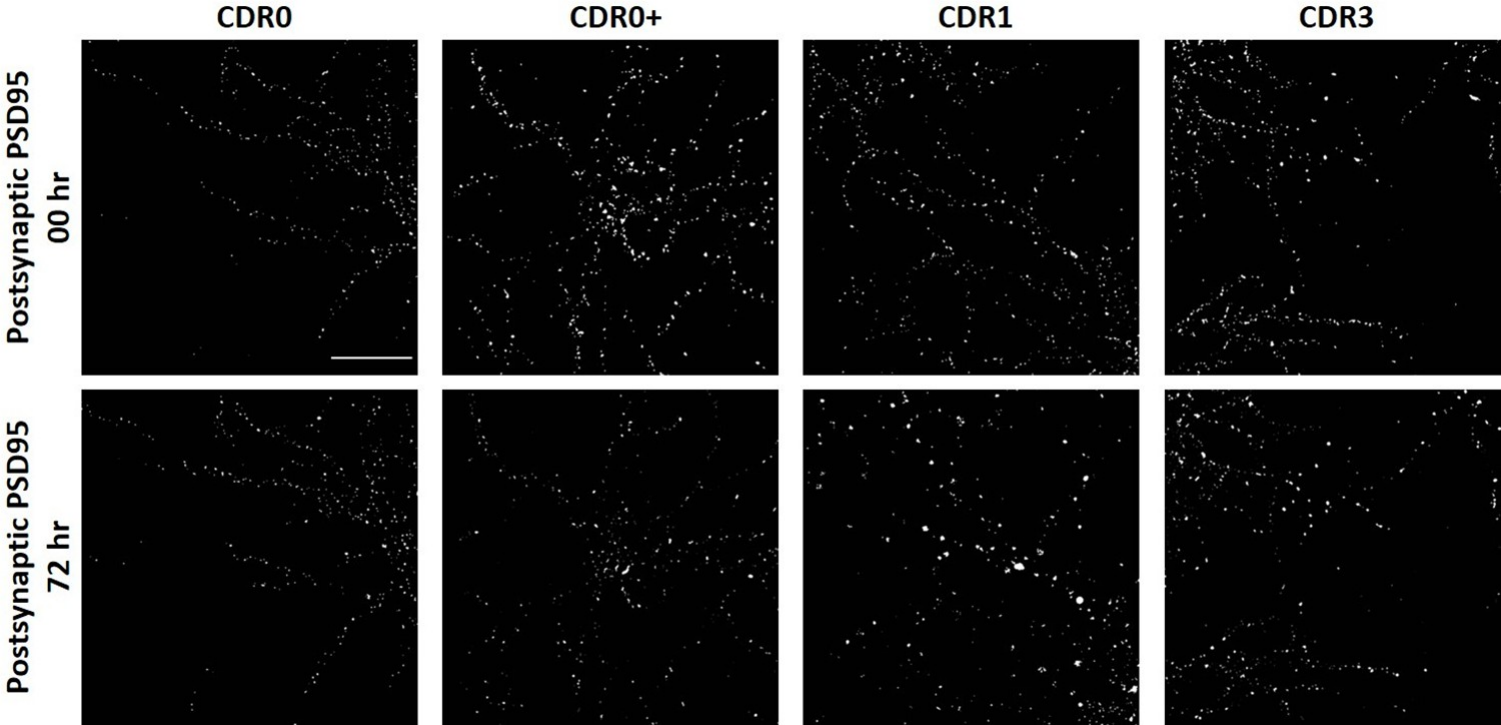
Representative images of PSD95 changes at baseline (00 hr) and after 72 hours incubation with homogenate Fraction 18 from CDR0, CDR0+, CDR1, and CDR3 groups. Mild loss of PSD95 was observed in CDR0 group, while severe loss of PSD95 was observed in CDR0+ and CDR1 groups. Scale bar = 50 μm.

HCS screening was performed on samples from a total of 29 subjects across four groups. Surprisingly, all four groups including CDR0 contained synaptotoxic activities in low molecular weight fractions (Figs 1I, 4 and 5, S2 and S3 Figs). However, the level of presynaptic VAMP2 loss in wells incubated for 72 hours with fractions from AD pathology positive samples (CDR0+, CDR1, and CDR3) were significantly higher than CDR0 samples by Dunnett’s multiple comparisons test (Fig 4A). CDR1 samples lysate fractions induced the most severe loss, followed by CDR0+ sample lysate fractions. Similar trends were observed in loss of postsynaptic PSD95, though most postsynaptic changes were not statistically significant by Dunnett’s multiple comparison tests (Figs 4B and 5). The loss of colocalized pre- and post-synaptic puncta was similar to the level of presynaptic VAMP2 loss: significant loss of colocalized pre and postsynaptic structures were identified only in fractions that caused significant pre and post synapse loss individually (F17 to F20) (Fig 4C). Similar results were obtained after 24 hours of exposure (S2 Fig). The detailed results of statistical comparison between sample groups using two-way ANOVA followed by Tukey’s multiple comparisons test have been presented in S1 and S2 Tables.

### Immunodepletion of Aβ and tau fails to rescue synapse loss

**Fig 6:**
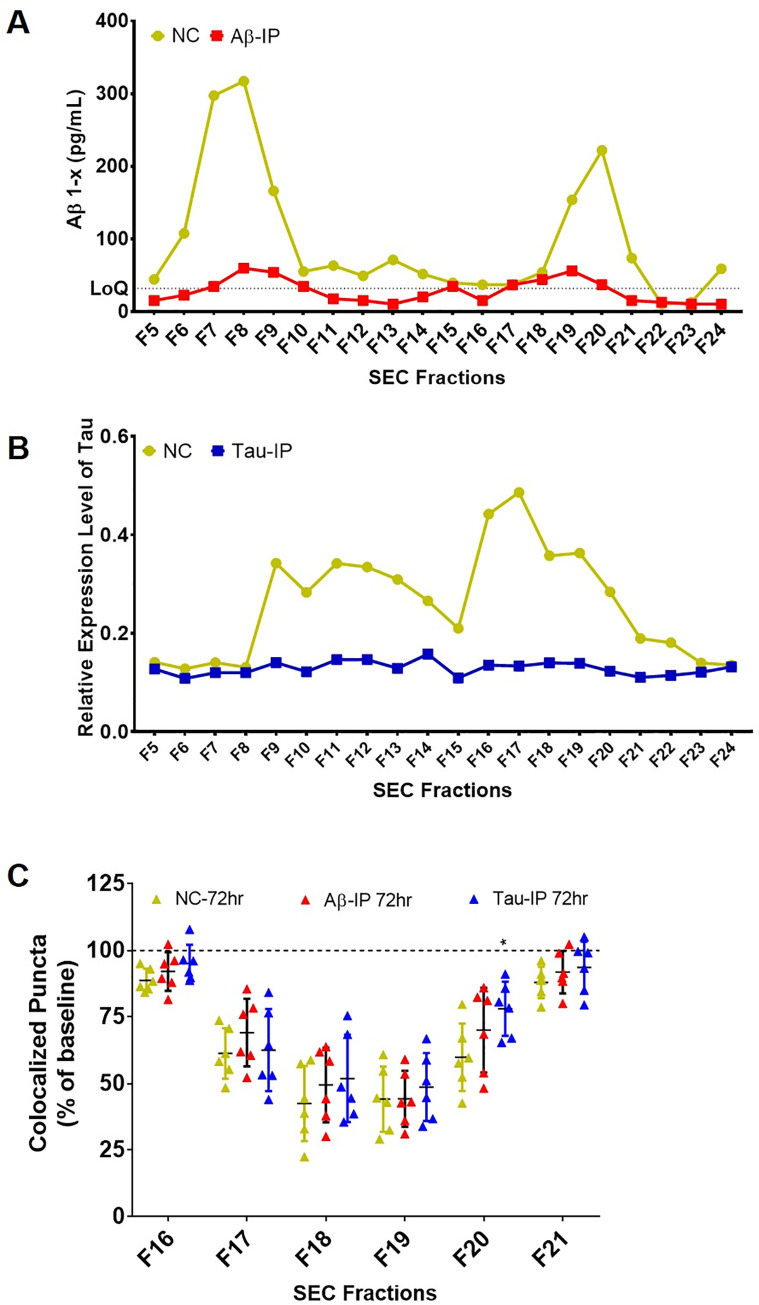
Comparison of the number of colocalized puncta between raw and Aβ and tau immunodepleted brain lysate fractions. (A, B) The efficiency of immunodepletion of Aβ and tau were evaluated by ELISA; (A) Sandwich ELISA of Aβ showed almost complete removal of Aβ; (B) Indirect ELISA of tau in each fraction showed a complete removal of tau in all fractions; (C) No significant difference in synaptotoxic activity was identified after immunodepletion of Aβ or tau; immunodepletions of Aβ and tau slightly decreased the extent of synapse loss in Fraction 16 to 21, however, the only significant different was that F20 in CDR3 homogenates showed reduced synapse loss following immunodepletion of tau.

We previously demonstrated that immunodepletion of Aβ from 3xTg-AD mouse brain lysates partially rescues synapse loss. Although not fully prevented by Aβ removal, these results indicate that some synapse loss is Aβ-dependent in this model. We find that the synaptotoxic activities in SEC separated human brain homogenates are both slightly larger than those in murine samples and larger than Aβ monomers. Interestingly, the most synaptotoxic fractions were among those with the highest tau concentrations, but tau levels were not well-correlated with synaptotoxic activity in that other fractions with high tau levels were not synaptotoxic. To assess whether synaptotoxic activities in human brain lysates are associated with Aβ or tau, we immunodepleted Aβ using HJ3.4 and HJ5.1 antibodies and separately immunodepleted tau using HJ8.7 and HJ9.3 antibodies followed by SEC separation of CDR0+ and CDR1 samples (n = 3 for each group). Almost all immunoreactive tau and Aβ were removed by immunodepletion as evaluated by ELISA (Fig 6A and 6B). The synaptic activities from fractions 16 to 21 were compared before and after the immunodepletions. Surprisingly, no significant differences were identified among control and depleted within factions 16–21 except for F20 with respect to tau depletion (Two-way ANOVA: F_(10,90)_ = 0.5277, *p =* 0.8664; Dunnett’s multiple comparisons test for F20: *p =* 0.0145, Fig 6C, S3 Table). Taken together, these results suggest that synaptotoxic activities in human brain lysates are Aβ and tau independent.

### Correlation of age and post-mortem interval with synaptotoxic activity

To determine whether synaptotoxic activities are related to age or post-mortem interval of the subjects, Pearson correlation coefficients between age and post-mortem interval vs. averaged percentage loss of colocalized synaptic puncta were calculated. Neither the age of subjects (R^2^ = 0.0430 and *p* = 0.2804) nor the post-mortem interval of the subjects (R^2^ = 0.0436 and *p* = 0.2770) were significantly correlated with synaptotoxic activities (S4 Fig).

## Discussion

AD is a slowly progressive neurodegenerative disease characterized by aggregation of Aβ and tau. Although the pathophysiological roles of Aβ and tau have been widely explored, the pathogenesis of AD remains inadequately understood. Evidence demonstrates that synapse loss, rather than Aβ plaques, NFTs, or neuronal loss, is most tightly correlated to dementia in AD [18, 35]. Numerous studies demonstrated that many individuals are able to remain cognitively normal in the presence of Aβ plaques [3, 4]. In an attempt to understand the relationship between synaptotoxicity and Aβ, we performed an unbiased screen for synaptotoxic activities in the brain homogenates from normal, normal with AD pathology, and AD subjects. We found synaptotoxic activities in brain homogenates that did not appear to be related to either Aβ or tau.

Using our recently developed HCS assay, we compared the synaptotoxic activities in SEC separated human frontal cortex homogenates from 29 individuals. Synaptotoxic activities were identified in low molecular weight SEC fractions from all individuals. Remarkably, this effect was not only observed in CDR1 and CDR3 AD patients, but also CDR0+ and CDR0 non-demented controls. Furthermore, samples from CDR0+ and CDR1 groups exhibited the most severe synapse loss, outpacing even CDR3 AD patients. This finding suggests that such synaptotoxic factors are present before clinical dementia, consistent with the earl onset of synapse loss in AD [35]. Synaptotoxic activities were also present in CDR0 normal controls, though the effects were smaller. This potentially reveals a synaptotoxic environment that also develops with healthy aging, albeit with reduced activity. Alternatively, it is possible that, individuals in the CDR0 groups may possess compensatory mechanisms that rescue or protect synapses from toxicity *in vivo* which were not captured in our cell-culture based assay.

Interestingly, homogenates from CDR0+ individuals showed synaptotoxic activity that was similar to that of homogenates from the CDR1 group and much worse than CDR0 normal individuals, suggesting the AD-related synaptotoxic effect also occurs in CDR0+ individuals. This result may be concordant with the findings of studies using ^18^F-Fludeoxyglucose Positron Emission Tomography which indicated that loss of neuronal function could be detected decades before the onset of AD [36]. The presence of synaptotoxic activity in CDR0+ individuals detected in our study potentially provides new evidence for pre-symptomatic cellular and molecular pathophysiology, though it remains unclear whether these CDR0+ individuals have preclinical AD or may also have compensatory factors that protect them from dementia. The identified synaptotoxic effect in these unique individuals may provide new understanding of the progression of AD and AD pathophysiology. On the other hand, we also found that homogenates from CDR1 individuals showed a higher synaptotoxic activity than homogenates from CDR3 cases, indicating the synaptotoxic effect is more active in early stage AD compared with late stage AD.

The synaptotoxic effect of human brain homogenates seem independent of soluble Aβ and tau. It is notable that numerous studies have demonstrated that soluble Aβ oligomers are the main neurotoxic component in extracts from AD brain tissues [20–22, 37–39]. While most of these studies focused on either synaptic plasticity (especially long-term potentiation) or neuronal toxicity, our assay focused on the synaptic loss by measuring the number of pre-, post-, and colocalized synapses before and after the treatment. On the other hand, in this study, we aimed to use an unbiased screening approach to identify the potential Aβ dependent and Aβ independent synaptotoxic substances in extracts from AD cases, cognitively normal individuals with AD pathology, and healthy controls. Therefore, instead of using previously identified Aβ or tau enriched fractions, we used the whole tissue extracts and only separated by SEC in ‘Neurobasal Salt’ solution. It is possible that the amount of previously identified Aβ oligomers in our final SEC fractioned samples was relatively low compared with previous studies and therefore was below the detection limit of our assay. In addition, we only accessed soluble portions of brain homogenates and, therefore, synaptotoxic activities associated with plaques, NFTs or other insoluble forms of Aβ and tau were not assessed. It is also possible that the specific epitopes of Aβ or tau responsible for synaptic toxicity are missing or protected by other proteins that prevent them from immunodepletion. In fact, multiple forms of soluble Aβ or tau may be present in SEC fraction with middle range molecular weight. For example, we (Fig 3B) and others [34] have identified anti-Aβ immunoreactive signals at relatively low levels in the middle range SEC fractions. Meanwhile, both ELISA and Western blot studies using various anti-tau antibodies showed different expression pattern between normal and AD subjects (Fig 2E and 2F) [40, 41]. Preliminary results from our group have highlighted the heterogeneity and complexity of native structures of soluble Aβ aggregates [42]. Regardless of the exact mechanisms, the dissociation between Aβ immunoreactivity from synaptotoxicity could hypothetically be related to the failure of the majority of antibody based immunotherapies targeting Aβ. Although many reasons have been put forward for the failure of these clinical trials [43], there is increasing evidence to support doubts about whether Aβ is the primary cause of the most common late onset, non-autosomal dominant forms of AD [44, 45].

Several limitations of this study should be noted. First, our assay measures synapses in a chemical defined medium and may not reflect the *in vivo* conditions in brain including the presence of astrocytes and microglia. Second, our assay focuses on the number of pre-, post-, and colocalized synaptic puncta without the assessment of synaptic plasticity or other functional activity. In fact, several studies have found that soluble tau aggregates [46], low molecular weight rather than high molecular weight Aβ oligomers [47], low molecular weight Aβ oligomers and Aβ monomers [48] can inhibit synaptic plasticity. Third, although no to very little immunoreactive Aβ or tau was left after immunodepletion, it is possible that certain modified or truncated Aβ or tau species or fragments may still remain in the samples. Extensive immunodepletion of tau by a combination of different anti-tau antibodies is needed for future studies. Fourth, as a pilot study for unbiased screening of synaptotoxic activities in control and AD human brain homogenates, we have screened over several hundreds of SEC fractions, however, the number of total subjects in each sample group was relatively small. Additional studies involving a wider variety of brain samples from different brain regions will be important. Fifth, the identified synaptotoxic activities could be artifactual. It is possible that synaptotoxic substances were released during the homogenization and protein extraction of the human brain tissue. To minimize potential artifacts, we used a Dounce tissue grinder to avoid high-speed turbulence, mechanical shearing, and potential foaming from rotor style homogenizers. Furthermore, compared with most previous studies that used PBS or TBS based buffers, a ‘Neurobasal Salt’ buffer (see details in method section) was used in our study to provide a more physiological condition as well as to minimize the introduction of extra salts and chemicals to downstream culture assays. Finally, additional work beyond the scope of the current communication will be required to identify the molecular natures of the synaptotoxic substances themselves.

In summary, we compared the synaptotoxic activities in SEC separated human frontal cortex homogenates from normal, normal with AD pathology, early and late stage AD individuals. Severe synaptotoxic activities were identified in CDR0+ and CDR1 samples, and the identified synaptotoxic activities seemed independent to Aβ and tau. Our results from CDR0+ individuals provided a new understanding of the relationship between AD pathology and AD pathogenesis.

## Supporting information

S1 Fig Scatterplots of individual levels Tau among control and AD patients. All values were measured by indirect ELISA, all samples were measured triplicated. One-way ANOVA followed by Tukey’s multiple comparisons test were used for all measurements (*** p ≤ 0.001, * p ≤ 0.05, error bar indicates S.D.). (A) Indirect ELISA using the HJ8.7 (anti-tau118-122 AAGHV [30]) antibody revealed significantly higher tau in the CDR0 group compared with all AD pathology positive samples (F(3,25) = 3.751, p = 0.0237; CDR0 vs CDR0+: p = 0.0484, CDR0 vs CDR1: p = 0.0005, and CDR0 vs CDR3: p = 0.0002); (B) No difference was found when using the HJ9.3 (anti-tau589-598 GGKVQIINKK, [1]) antibody (F(3,25) = 1.004, p = 0.4075; (C) Indirect ELISA using an anti-phosphorylated tau (phospho T205) antibody revealed a significantly higher level in the CDR0 group compared with CDR1 (F(3,25) = 3.448, p = 0.0318; CDR0 vs CDR1: p = 0.0408).
(TIF)

S2 Fig Scatterplots of individual levels of synaptotoxic activities in each SEC fractions from control and AD patient groups at 24 hours. Two-way ANOVA followed by Dunnett’s multiple comparison test between CDR0 and other groups were used for all measurements (**** p ≤ 0.0001, *** p ≤ 0.001, ** p ≤ 0.01, * p ≤ 0.05, error bar indicates SEM) (A) Comparison of presynaptic VAMP2 synaptotoxic activities among control and AD patient groups at 24 hours; severe VAMP2 loss was found in wells incubated with lysate fractions 17 to 20; (B) Comparison of postsynaptic PSD95 synaptotoxic activities among control and AD patient groups at 24 hours. Loss of PSD95 post synaptic puncta was found in wells incubated with lysate fractions 17 to 20. (C) Comparison of colocalized pre and post synaptic puncta among control and AD patient groups at 24 hours; loss of colocalized synaptic puncta was found in wells incubated with fractions 17 to 20.
(TIF)

S3 Fig Scatterplots of individual levels of synaptotoxic activities in each SEC fractions from control and AD case groups at 72 hours. Two-way ANOVA followed by Dunnett’s multiple comparison test between CDR0 and other groups were used for all measurements (**** p ≤ 0.0001, *** p ≤ 0.001, ** p ≤ 0.01, * p ≤ 0.05, error bar indicates SEM) (A) Comparison of presynaptic VAMP2 synaptotoxic activities among control and AD case groups at 72 hours; significant VAMP2 loss were found in wells incubated with lysate fractions 17 to 20; several fractions from CDR0+ and CDR1 showed statistically more VAMP2 loss than from CDR0; (B) Comparison of postsynaptic PSD95 synaptotoxic activities among control and AD case groups at 72 hours. Loss of PSD95 post synaptic puncta was found in wells incubated with lysate fractions 17 to 20. Fraction 18 from CDR0+ and CDR1 brain homogenates caused more PSD95 postsynapse loss than fraction 18 from CDR0 brains. Fraction 19 from CDR3 brain homogenates caused more PSD95 postsynapse loss than fraction 19 from CDR brains. (C) Comparison of colocalized pre and post synaptic puncta among control and AD case groups at 72 hours; loss of colocalized synaptic puncta were found in wells incubated with fractions 17 to 20. Fractions 17–19 from CDR0+ and CDR1 brain homogenates caused more colocalized synaptic puncta loss than comparable fractions from CDR0 brains.
(TIF)

S4 Fig Correlation of age and post-mortem interval with synaptotoxic activity. (A) Pearson correlation coefficients was calculated between average synaptotoxic activities of SEC fraction 16 to 20 and the age of each subjects. No significant correlation was found between synaptotoxic activities and the age of each subject with R2 = 0.0430 and p = 0.2804; (B) Pearson correlation coefficients between synaptotoxic activities and post-mortem interval was also not significant with R2 = 0.0436 and p = 0.2770.
(TIF)

S1 Table Adjusted P value of Tukey’s multiple comparisons test on synaptic puncta count between sample groups at 72 hours. (DOCX)

S2 Table Adjusted P value of Tukey’s multiple comparisons test on synaptic puncta count between sample groups at 24 hours. (DOCX)

S3 Table Adjusted P value of Dunnett’s multiple comparisons test on synaptic puncta count between control and immunodepletion samples at 72 hours. (DOCX)
