# Supplementary figures and images for: Unbiased high-content screening reveals Aβ- and tau-independent synaptotoxic activities in human brain homogenates from Alzheimer’s patients and high-pathology controls
Source: PLoS One. 2021 Nov 8;16(11):e0259335. doi: 10.1371/journal.pone.0259335 (PMC8575250; doi:10.1371/journal.pone.0259335)

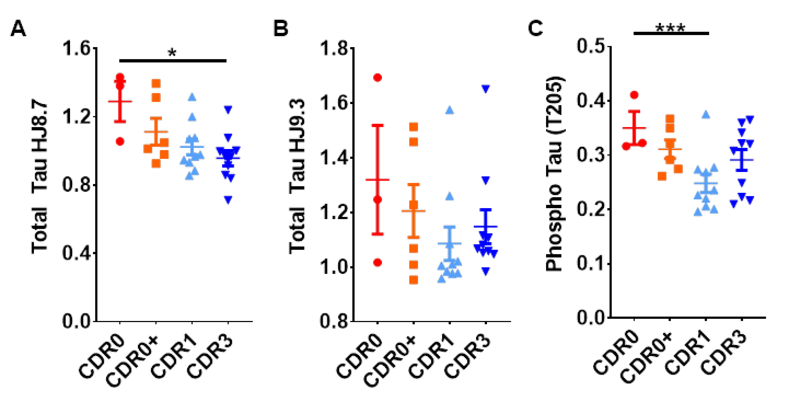

Supplement: S1 Fig — All values were measured by indirect ELISA, all samples were measured triplicated. One-way ANOVA followed by Tukey’s multiple comparisons test were used for all measurements (*** p ≤ 0.001, * p ≤ 0.05, error bar indicates S.D.). (A) Indirect ELISA using the HJ8.7 (anti-tau118-122 AAGHV [30]) antibody revealed significantly higher tau in the CDR0 group compared with all AD pathology positive samples (F(3,25) = 3.751, p = 0.0237; CDR0 vs CDR0+: p = 0.0484, CDR0 vs CDR1: p = 0.0005, and CDR0 vs CDR3: p = 0.0002); (B) No difference was found when using the HJ9.3 (anti-tau589-598 GGKVQIINKK, [1]) antibody (F(3,25) = 1.004, p = 0.4075; (C) Indirect ELISA using an anti-phosphorylated tau (phospho T205) antibody revealed a significantly higher level in the CDR0 group compared with CDR1 (F(3,25) = 3.448, p = 0.0318; CDR0 vs CDR1: p = 0.0408). (TIF) [file pone.0259335.s001.tif]

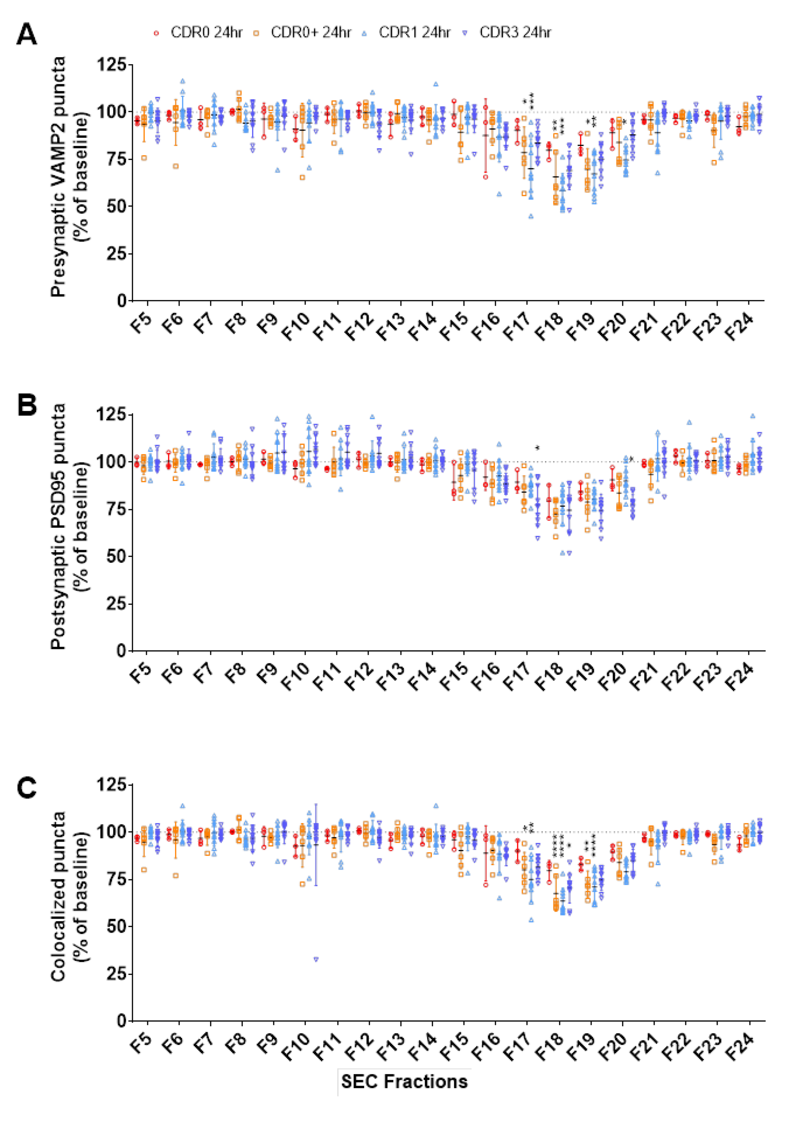

Supplement: S2 Fig — Two-way ANOVA followed by Dunnett’s multiple comparison test between CDR0 and other groups were used for all measurements (**** p ≤ 0.0001, *** p ≤ 0.001, ** p ≤ 0.01, * p ≤ 0.05, error bar indicates SEM) (A) Comparison of presynaptic VAMP2 synaptotoxic activities among control and AD patient groups at 24 hours; severe VAMP2 loss was found in wells incubated with lysate fractions 17 to 20; (B) Comparison of postsynaptic PSD95 synaptotoxic activities among control and AD patient groups at 24 hours. Loss of PSD95 post synaptic puncta was found in wells incubated with lysate fractions 17 to 20. (C) Comparison of colocalized pre and post synaptic puncta among control and AD patient groups at 24 hours; loss of colocalized synaptic puncta was found in wells incubated with fractions 17 to 20. (TIF) [file pone.0259335.s002.tif]

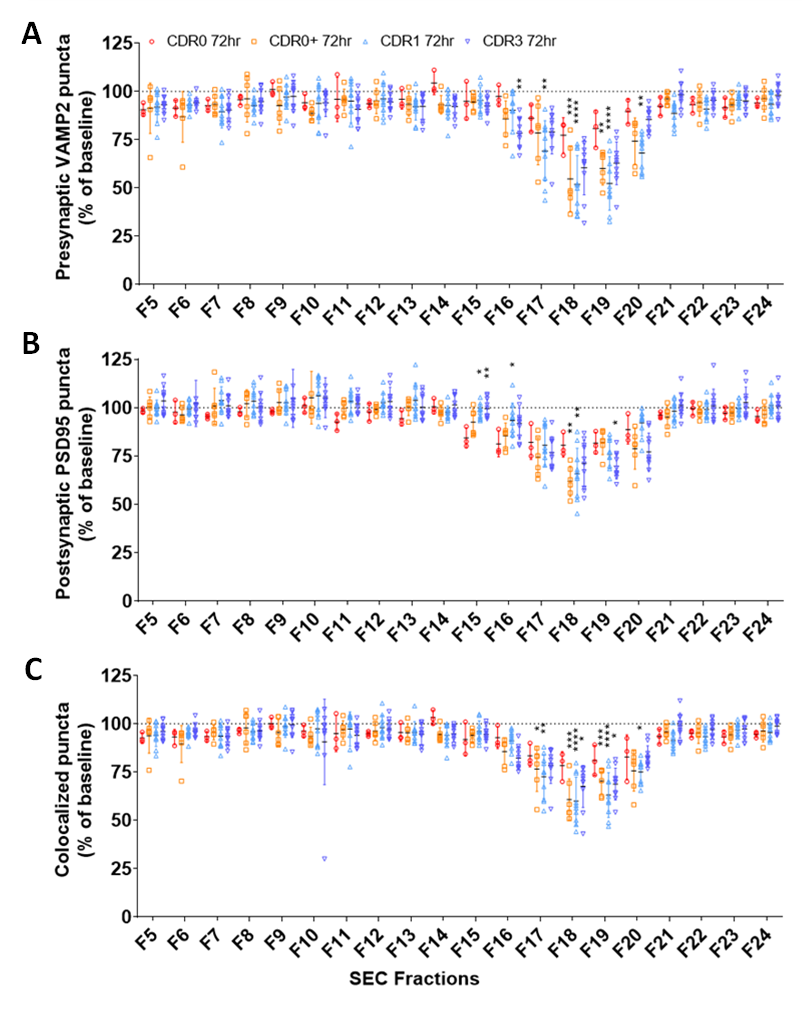

Supplement: S3 Fig — Two-way ANOVA followed by Dunnett’s multiple comparison test between CDR0 and other groups were used for all measurements (**** p ≤ 0.0001, *** p ≤ 0.001, ** p ≤ 0.01, * p ≤ 0.05, error bar indicates SEM) (A) Comparison of presynaptic VAMP2 synaptotoxic activities among control and AD case groups at 72 hours; significant VAMP2 loss were found in wells incubated with lysate fractions 17 to 20; several fractions from CDR0+ and CDR1 showed statistically more VAMP2 loss than from CDR0; (B) Comparison of postsynaptic PSD95 synaptotoxic activities among control and AD case groups at 72 hours. Loss of PSD95 post synaptic puncta was found in wells incubated with lysate fractions 17 to 20. Fraction 18 from CDR0+ and CDR1 brain homogenates caused more PSD95 postsynapse loss than fraction 18 from CDR0 brains. Fraction 19 from CDR3 brain homogenates caused more PSD95 postsynapse loss than fraction 19 from CDR brains. (C) Comparison of colocalized pre and post synaptic puncta among control and AD case groups at 72 hours; loss of colocalized synaptic puncta were found in wells incubated with fractions 17 to 20. Fractions 17–19 from CDR0+ and CDR1 brain homogenates caused more colocalized synaptic puncta loss than comparable fractions from CDR0 brains. (TIF) [file pone.0259335.s003.tif]

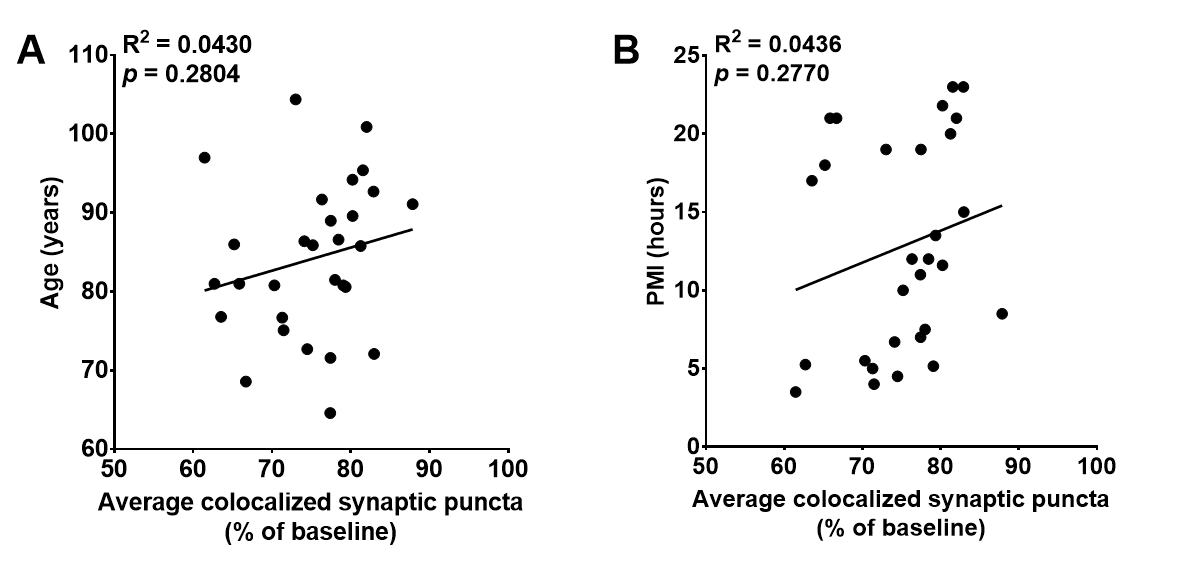

Supplement: S4 Fig — (A) Pearson correlation coefficients was calculated between average synaptotoxic activities of SEC fraction 16 to 20 and the age of each subjects. No significant correlation was found between synaptotoxic activities and the age of each subject with R2 = 0.0430 and p = 0.2804; (B) Pearson correlation coefficients between synaptotoxic activities and post-mortem interval was also not significant with R2 = 0.0436 and p = 0.2770. (TIF) [file pone.0259335.s004.tif]
