# Supplementary material for: Unbiased high-content screening reveals Aβ- and tau-independent synaptotoxic activities in human brain homogenates from Alzheimer’s patients and high-pathology controls
Source: PLoS One. 2021 Nov 8;16(11):e0259335. doi: 10.1371/journal.pone.0259335 (PMC8575250; doi:10.1371/journal.pone.0259335)
Supplement: S1 Table — (DOCX) [file pone.0259335.s005.docx]

# S1 Table. Adjusted P value of Tukey's multiple comparisons test on synaptic puncta count between sample groups at 72 hours.

| **Pre-synaptic VAMP2** | | | | | | |
| --- | --- | --- | --- | --- | --- | --- |
| **SEC Fractions** | **CDR0vsCDR0+** | **CDR0vsCDR1** | **CDR0vsCDR3** | **CDR0+vsCDR1** | **CDR0+vsCDR3** | **CDR1vsCDR3** |
| **F5** | 0.999 | 0.994 | 0.967 | 0.999 | 0.977 | 0.990 |
| **F6** | 0.879 | 0.992 | 0.960 | 0.514 | 0.348 | 0.989 |
| **F7** | 0.999 | 0.945 | 0.980 | 0.818 | 0.911 | 0.995 |
| **F8** | 1.000 | 0.953 | 0.998 | 0.866 | 0.988 | 0.955 |
| **F9** | 0.515 | 0.904 | 0.925 | 0.740 | 0.696 | 1.000 |
| **F10** | 0.798 | 0.999 | 0.999 | 0.641 | 0.615 | 1.000 |
| **F11** | 0.999 | 0.998 | 0.797 | 0.999 | 0.680 | 0.689 |
| **F12** | 0.994 | 0.968 | 0.998 | 0.995 | 0.999 | 0.973 |
| **F13** | 0.974 | 0.907 | 0.917 | 0.993 | 0.995 | 0.999 |
| **F14** | 0.253 | 0.161 | 0.136 | 0.999 | 0.996 | 0.999 |
| **F15** | 0.999 | 1.000 | 0.976 | 0.998 | 0.969 | 0.885 |
| **F16** | 0.218 | 0.579 | **0.004** | 0.741 | 0.344 | **0.011** |
| **F17** | 0.350 | **0.003** | 0.520 | 0.131 | 0.954 | **0.011** |
| **F18** | **0.001** | **0.000** | 0.152 | 0.907 | **0.043** | **0.001** |
| **F19** | **0.004** | **0.000** | 0.098 | 0.161 | 0.303 | **0.000** |
| **F20** | 0.177 | **0.006** | 0.997 | 0.496 | 0.058 | **0.000** |
| **F21** | 0.923 | 0.913 | 0.724 | 0.324 | 0.966 | 0.061 |
| **F22** | 0.998 | 0.977 | 0.976 | 0.879 | 0.991 | 0.623 |
| **F23** | 0.995 | 0.896 | 0.921 | 0.943 | 0.965 | 1.000 |
| **F24** | 0.986 | 0.999 | 0.897 | 0.918 | 0.975 | 0.607 |
| **Post-synaptic PSD95** | | | | | | |
| **SEC**  **Fractions** | **CDR0vsCDR0+** | **CDR0vsCDR1** | **CDR0vsCDR3** | **CDR0+vsCDR1** | **CDR0+vsCDR3** | **CDR1vsCDR3** |
| **F5** | 0.986 | 0.992 | 0.718 | 1.000 | 0.820 | 0.672 |
| **F6** | 0.993 | 0.989 | 0.791 | 0.866 | 0.408 | 0.807 |
| **F7** | 0.741 | 0.336 | 0.662 | 0.876 | 1.000 | 0.861 |
| **F8** | 0.849 | 0.673 | 0.965 | 0.988 | 0.957 | 0.770 |
| **F9** | 0.809 | 0.796 | 0.503 | 0.999 | 0.945 | 0.895 |
| **F10** | 0.867 | 0.721 | 0.835 | 0.992 | 0.999 | 0.990 |
| **F11** | 0.523 | 0.141 | 0.216 | 0.822 | 0.933 | 0.989 |
| **F12** | 0.995 | 0.759 | 0.683 | 0.790 | 0.696 | 0.998 |
| **F13** | 0.694 | 0.220 | 0.645 | 0.776 | 0.999 | 0.687 |
| **F14** | 0.939 | 0.924 | 0.998 | 0.999 | 0.732 | 0.626 |
| **F15** | 0.406 | **0.028** | **0.011** | 0.478 | 0.267 | 0.971 |
| **F16** | 0.857 | 0.068 | 0.139 | 0.173 | 0.351 | 0.969 |
| **F17** | 0.450 | 0.990 | 0.704 | 0.359 | 0.914 | 0.668 |
| **F18** | **0.002** | **0.015** | 0.214 | 0.741 | 0.082 | 0.393 |
| **F19** | 1.000 | 0.630 | 0.077 | 0.366 | **0.009** | 0.283 |
| **F20** | 0.242 | 0.890 | 0.090 | **0.003** | 0.974 | **0.000** |
| **F21** | 0.998 | 0.975 | 0.736 | 0.855 | 0.399 | 0.812 |
| **F22** | 0.984 | 1.000 | 0.991 | 0.977 | 0.815 | 0.947 |
| **F23** | 0.999 | 0.965 | 0.676 | 0.948 | 0.516 | 0.773 |
| **F24** | 0.997 | 0.654 | 0.691 | 0.635 | 0.683 | 1.000 |
| **Colocalized-synaptic puncta** | | | | | | |
| **SEC**  **Fractions** | **CDR0vsCDR0+** | **CDR0vsCDR1** | **CDR0vsCDR3** | **CDR0+vsCDR1** | **CDR0+vsCDR3** | **CDR1vsCDR3** |
| **F5** | 0.997 | 0.9919 | 0.9069 | 0.9998 | 0.9379 | 0.9405 |
| **F6** | 0.9039 | 0.9889 | 0.9096 | 0.5388 | 0.2823 | 0.9557 |
| **F7** | 0.9805 | 1.0000 | 1.0000 | 0.9595 | 0.9546 | 1.0000 |
| **F8** | 0.9913 | 0.9996 | 1.0000 | 0.9498 | 0.977 | 0.9988 |
| **F9** | 0.8225 | 0.9912 | 0.9995 | 0.8493 | 0.7238 | 0.9932 |
| **F10** | 0.9536 | 0.9944 | 0.6746 | 0.7284 | 0.8902 | 0.1803 |
| **F11** | 0.9846 | 0.9654 | 0.9973 | 0.9995 | 0.8819 | 0.7593 |
| **F12** | 0.9927 | 0.915 | 0.9701 | 0.9688 | 0.9974 | 0.9906 |
| **F13** | 1.0000 | 1.0000 | 0.9974 | 1.0000 | 0.9973 | 0.9924 |
| **F14** | 0.3444 | 0.2433 | 0.3244 | 0.9996 | 0.9995 | 0.9948 |
| **F15** | 0.9792 | 0.8284 | 0.9502 | 0.9468 | 0.9989 | 0.9661 |
| **F16** | 1.0000 | 0.8341 | 1.0000 | 0.7276 | 1.0000 | 0.6199 |
| **F17** | **0.0286** | **0.0074** | 0.548 | 0.9907 | 0.1479 | 0.0276 |
| **F18** | **0.0008** | **0.0000** | 0.0516 | **0.0018** | 0.1978 | **0.0000** |
| **F19** | **0.0003** | **0.0000** | **0.03** | **0.0474** | 0.1598 | **0.0000** |
| **F20** | 0.3979 | **0.0308** | 0.7783 | 0.5212 | 0.7848 | **0.0401** |
| **F21** | 0.9924 | 0.8774 | 0.6567 | 0.5408 | 0.6969 | **0.0273** |
| **F22** | 1.0000 | 0.9852 | 0.9749 | 0.9613 | 0.9607 | 0.6681 |
| **F23** | 0.9967 | 0.8954 | 0.8387 | 0.9289 | 0.866 | 0.9976 |
| **F24** | 0.9865 | 0.9964 | 0.8149 | 0.9977 | 0.9128 | 0.7631 |
