# Supplementary material for: Unbiased high-content screening reveals Aβ- and tau-independent synaptotoxic activities in human brain homogenates from Alzheimer’s patients and high-pathology controls
Source: PLoS One. 2021 Nov 8;16(11):e0259335. doi: 10.1371/journal.pone.0259335 (PMC8575250; doi:10.1371/journal.pone.0259335)
Supplement: S2 Table — (DOCX) [file pone.0259335.s006.docx]

# S2 Table. Adjusted P value of Tukey's multiple comparisons test on synaptic puncta count between sample groups at 24 hours.

| **Pre-synaptic VAMP2** | | | | | | |
| --- | --- | --- | --- | --- | --- | --- |
| **SEC Fractions** | **CDR0vsCDR0+** | **CDR0vsCDR1** | **CDR0vsCDR3** | **CDR0+vsCDR1** | **CDR0+vsCDR3** | **CDR1vsCDR3** |
| **F5** | 0.988 | 0.885 | 0.999 | 0.518 | 0.965 | 0.728 |
| **F6** | 0.926 | 0.972 | 0.999 | 0.499 | 0.899 | 0.847 |
| **F7** | 0.997 | 0.969 | 0.993 | 0.990 | 1.000 | 0.994 |
| **F8** | 0.997 | 0.694 | 0.816 | 0.332 | 0.491 | 0.989 |
| **F9** | 0.999 | 0.993 | 0.991 | 0.987 | 0.980 | 0.835 |
| **F10** | 1.000 | 0.936 | 0.645 | 0.818 | 0.375 | 0.825 |
| **F11** | 0.965 | 0.975 | 0.973 | 1.000 | 1.000 | 0.999 |
| **F12** | 0.998 | 0.999 | 0.619 | 0.999 | 0.560 | 0.390 |
| **F13** | 0.792 | 0.910 | 0.984 | 0.976 | 0.851 | 0.969 |
| **F14** | 0.992 | 0.996 | 0.999 | 1.000 | 0.997 | 0.999 |
| **F15** | 0.399 | 0.989 | 0.968 | 0.303 | 0.401 | 0.997 |
| **F16** | 0.937 | 0.999 | 0.999 | 0.767 | 0.766 | 0.999 |
| **F17** | **0.040** | **0.001** | 0.522 | 0.653 | 0.226 | **0.003** |
| **F18** | **0.006** | **0.000** | 0.231 | 0.230 | 0.166 | **0.000** |
| **F19** | **0.043** | **0.013** | 0.458 | 0.993 | 0.306 | 0.094 |
| **F20** | 0.840 | 0.054 | 0.998 | 0.151 | 0.807 | **0.003** |
| **F21** | 0.999 | 0.593 | 0.931 | 0.427 | 0.833 | **0.033** |
| **F22** | 0.999 | 0.994 | 0.997 | 0.991 | 0.992 | 0.904 |
| **F23** | 0.547 | 0.943 | 1.000 | 0.689 | 0.320 | 0.896 |
| **F24** | 0.814 | 0.731 | 0.667 | 1.000 | 0.995 | 0.999 |
| **Post-synaptic PSD95** | | | | | | |
| **SEC**  **Fractions** | **CDR0vsCDR0+** | **CDR0vsCDR1** | **CDR0vsCDR3** | **CDR0+vsCDR1** | **CDR0+vsCDR3** | **CDR1vsCDR3** |
| **F5** | 0.983 | 0.999 | 0.999 | 0.960 | 0.903 | 0.996 |
| **F6** | 0.997 | 0.999 | 0.994 | 0.988 | 0.930 | 0.988 |
| **F7** | 0.999 | 0.842 | 0.926 | 0.666 | 0.818 | 0.990 |
| **F8** | 0.998 | 0.994 | 0.999 | 1.000 | 0.996 | 0.981 |
| **F9** | 0.972 | 0.911 | 0.880 | 0.472 | 0.411 | 0.999 |
| **F10** | 0.946 | 0.234 | 0.153 | 0.351 | 0.217 | 0.989 |
| **F11** | 0.894 | 0.683 | 0.259 | 0.970 | 0.520 | 0.712 |
| **F12** | 0.979 | 0.973 | 0.902 | 0.690 | 0.485 | 0.982 |
| **F13** | 0.999 | 0.994 | 0.961 | 0.979 | 0.901 | 0.986 |
| **F14** | 0.997 | 0.960 | 0.969 | 0.985 | 0.991 | 0.999 |
| **F15** | 0.894 | 0.202 | 0.560 | 0.414 | 0.898 | 0.764 |
| **F16** | 0.906 | 0.999 | 0.880 | 0.686 | 0.999 | 0.561 |
| **F17** | 0.727 | 0.966 | 0.053 | 0.843 | 0.257 | **0.012** |
| **F18** | 0.536 | 0.942 | 0.735 | 0.679 | 0.951 | 0.907 |
| **F19** | 0.698 | 0.841 | 0.158 | 0.973 | 0.641 | 0.248 |
| **F20** | 0.547 | 0.999 | **0.037** | 0.343 | 0.374 | **0.001** |
| **F21** | 0.702 | 0.943 | 0.999 | 0.125 | 0.334 | 0.933 |
| **F22** | 0.901 | 0.996 | 0.962 | 0.912 | 0.988 | 0.981 |
| **F23** | 0.999 | 0.989 | 0.981 | 0.978 | 0.964 | 1.000 |
| **F24** | 0.965 | 0.419 | 0.668 | 0.559 | 0.853 | 0.937 |
| **Colocalized-synaptic puncta** | | | | | | |
| **SEC**  **Fractions** | **CDR0vsCDR0+** | **CDR0vsCDR1** | **CDR0vsCDR3** | **CDR0+vsCDR1** | **CDR0+vsCDR3** | **CDR1vsCDR3** |
| **F5** | 0.9853 | 0.9419 | 1.000 | 0.6265 | 0.9461 | 0.8767 |
| **F6** | 0.9499 | 0.9855 | 1.000 | 0.6399 | 0.8955 | 0.9467 |
| **F7** | 0.9991 | 0.9404 | 0.9811 | 0.949 | 0.9908 | 0.9923 |
| **F8** | 0.9966 | 0.862 | 0.9026 | 0.5703 | 0.6497 | 0.9989 |
| **F9** | 0.9993 | 1.000 | 0.9738 | 0.9994 | 0.8908 | 0.9023 |
| **F10** | 0.9999 | 0.7721 | 0.9992 | 0.6759 | 0.9999 | 0.6147 |
| **F11** | 0.998 | 1.000 | 0.9988 | 0.9971 | 0.972 | 0.9929 |
| **F12** | 0.995 | 1.000 | 0.8715 | 0.9862 | 0.9195 | 0.6694 |
| **F13** | 0.8938 | 0.9356 | 0.9766 | 0.9962 | 0.9703 | 0.9938 |
| **F14** | 0.9983 | 1.000 | 1.000 | 0.998 | 0.9948 | 0.9998 |
| **F15** | 0.7305 | 0.9925 | 1.000 | 0.2902 | 0.4979 | 0.9749 |
| **F16** | 0.9142 | 0.9779 | 0.9462 | 0.981 | 0.9975 | 0.9963 |
| **F17** | **0.0215** | **0.0033** | 0.1012 | 0.9659 | 0.7365 | 0.3136 |
| **F18** | **0.0000** | **0.0000** | 0.0500 | 1.000 | **0.0028** | **0.0003** |
| **F19** | **0.0065** | **0.0000** | 0.1904 | 0.1659 | 0.2218 | **0.0000** |
| **F20** | 0.8659 | 0.3806 | 0.6734 | 0.7629 | 0.982 | 0.8992 |
| **F21** | 0.8054 | 0.4739 | 0.9977 | 0.9298 | 0.4577 | 0.0791 |
| **F22** | 0.9971 | 0.9938 | 1.000 | 1.000 | 0.9894 | 0.9737 |
| **F23** | 0.7309 | 0.985 | 1.000 | 0.7698 | 0.4628 | 0.9404 |
| **F24** | 0.848 | 0.6198 | 0.6343 | 0.9733 | 0.9782 | 1.000 |
