# Supplementary material for: Unbiased high-content screening reveals Aβ- and tau-independent synaptotoxic activities in human brain homogenates from Alzheimer’s patients and high-pathology controls
Source: PLoS One. 2021 Nov 8;16(11):e0259335. doi: 10.1371/journal.pone.0259335 (PMC8575250; doi:10.1371/journal.pone.0259335)
Supplement: S3 Table — (DOCX) [file pone.0259335.s007.docx]

# S3 Table. Adjusted P value of Dunnett's multiple comparisons test on synaptic puncta count between control and immunodepletion samples at 72 hours.

| **SEC Fractions** | **NC-72hr vs. Aβ-IP 72hr** | **NC-72hr vs. Tau-IP 72hr** |
| --- | --- | --- |
| **F16** | 0.8367 | 0.5355 |
| **F17** | 0.3975 | 0.9731 |
| **F18** | 0.4823 | 0.2759 |
| **F19** | 0.9997 | 0.7181 |
| **F20** | 0.2234 | **0.0145** |
| **F21** | 0.7911 | 0.6107 |
